# Tonic down-rolling and eccentric down-positioning of eyes under sevoflurane anesthesia without non-depolarizing muscle relaxant and its relationship with depth of anesthesia

**DOI:** 10.3389/fmed.2023.1029952

**Published:** 2023-06-15

**Authors:** Shweta Chaurasia, Shiv Lal Soni, Venkata Ganesh, Jagat Ram, Jaspreet Sukhija, Swati Chaurasia, Aastha Takkar

**Affiliations:** ^1^Department of Ophthalmology, Advanced Eye Centre, Post Graduate Institute of Medical Education and Research, Chandigarh, India; ^2^Department of Anaesthesiology, Post Graduate Institute of Medical Education and Research, Chandigarh, India; ^3^Department of Medicine, Sanjay Gandhi Memorial Hospital, New Delhi, India; ^4^Department of Neurology, Post Graduate Institute of Medical Education and Research, Chandigarh, India

**Keywords:** tonic down-rolling, minimal alveolar concentration, general anesthesia, downward eye movement, sevoflurane, laryngeal mask, eccentric eye positioning, non-depolarizing muscle relaxant

## Abstract

**Purpose:**

To analyze the relationship between eccentric downward eye movement/eccentric downward eye-positioning (EDEM/EDEP) encountered in patients undergoing ophthalmic surgeries and its return to a centralized position under general anesthesia (GA) with the depth of anesthesia (DOA).

**Methods:**

Patients undergoing ophthalmic surgeries (6 months-12 years) under sevoflurane anesthesia without non-depolarizing muscle relaxant (NDMR) who witnessed a sudden tonic EDEM/EDEP were both retrospectively (R-group) and prospectively (P-group) enrolled (ambispective study). R-group included data-points after induction (AI) till the time surgery lasted while P-group compiled data both during induction (DI) and AI. DOA in terms of MAC (minimum alveolar concentration) at the time of EDEM/EDEP and centralization of eyeball and their timings were noted and compared for both AI and DI data-points. Also, vertical eccentric eye positions were scored and correlated with MAC.

**Results:**

AI data included 22 (14R+8P) events and their mean MAC of EDEM/EDEP and centralization were 1.60 ± 0.25 and 1.18 ± 0.17 respectively (*p* = 0.000). DI data included 62 (P) cases and its mean MAC of EDEM/EDEP and centralization was 2.19 ± 0.43 and 1.39 ± 0.26 respectively (*p* = 0.000). Median (IQR) eye positions during down-positioning in 84 events was −3 (−3.9 to −2.5). It was preceded by an eccentric upward drift of eyes in 10/22 (6R+4P) AI cases. A strong negative correlation was seen between DOA and eccentric eye positions (*r* = −0.77, *p* = 0.000).

**Conclusions:**

Tonic down-rolling of eyes is not uncommon in children seen without NDMR with higher depths of sevoflurane anesthesia compared to point of centralization and fluctuations in DOA should be avoided to circumvent inadvertent complications during ocular surgery.

## 1. Introduction

Anesthetized patients go through Guedel's (1) stages of general anesthesia (GA). Guedel described how, as depth of anesthesia increases and the third stage of surgical anesthesia is reached, the extraocular muscles become flaccid and the eyeball movement ceases (1). But eye movements on the operating table are not uncommon in stage 3 anesthesia (2–4). Investigators found that during ocular surgery under GA, 18% of patients had eccentric eye movements (3). In a developing nation like ours with limited resources and high patients' volume, it is routine to perform short surgeries under sevoflurane anesthesia using a laryngeal mask without non-depolarizing muscle relaxant (NDMR). This newer drugs' rapid manipulation of anesthetic planes allows patients to move quickly between deeper and superficial planes (5). For an ophthalmologist, eccentric eye movements can pose a surgical challenge due to constriction of the field of surgery and sudden jerks which can cause inadvertent iatrogenic complications at different steps in the surgical procedures like cataracts, squints, etc. Slightly divergent and elevated eye positions during GA is a known finding (2, 6). But only a few studies have described fixed vertically deviated eye positions during ocular surgery under GA and discussed its definite relationship with anesthetic depth (2–4). According to a quantitative study, lighter planes of anesthesia without NDMR correlate with higher eye position, (4) which has been explained on the basis of natural Bell's phenomenon. Only a few authors have mentioned deeper levels of anesthesia with the down positioning of eyes (2, 4). To the best of our knowledge, there is no literature on the detailed documentation of tonic downward movement of the eyes and their eccentric positioning in down gaze in their relation to anesthetic depth.

So, we here aimed to investigate relationship DOA (in terms of MAC) with the abrupt downward eccentric eye movement/eccentric down-positioning (EDEM/EDEP) and its return to normal centralized position under sevoflurane in the absence of NDMR. Also, we aimed to evaluate their timings and correlate eye positions (eye score) with fluctuation in DOA. This study aims to raise awareness of this not uncommon ocular finding in children under SA that has important consequences in ophthalmic surgery when done without NDMR. Using our study's findings, we hope to evaluate the literature and gain insight into the intricacy of neuronal processes in subcortical regions mediating tonic eye movements under GA.

## 2. Materials and methods

### 2.1. Study type

We experienced EDEM/EDEP during surgery in a few retrospective cases, so we decided to prospectively recruit subjects and follow them from induction to end of surgery to witness sudden down-rolling events. Data-points during induction were included in prospective series to understand the relationship of eye movements with different DOA as maximum flow of sevoflurane and its fluctuation occur during induction. So, this was an ambispective observational study which was approved by our Institutional Ethics committee (retrospective IEC NK/7068/Study/068; prospective IEC NK/5860/Study/542 with CTRI no 2021/2021/10/037578S). This adhered to the tenets of the Declaration of Helsinki and written informed consent was obtained from all patients' parents or guardians regarding their study participation.

### 2.2. Study population

#### 2.2.1. Retrospective

Operative records of pediatric patients aged 6 months to 12 years of age who underwent surgeries (operated by single surgeon SC) under GA with a supraglottic airway device without NDMR between January 2018 and August 2021 were retrospectively reviewed from the recorded intra-operative data base of ophthalmic surgeries (cataract/squint/botulinum toxin) and cases in which vertically downward movement was witnessed intra-operatively by the ophthalmic surgeon with or without an accompanying upward movement under direct observation during surgery were included in our study.

#### 2.2.2. Prospective

Patients aged 6 months to 12 years of age who underwent squint surgery/botulinum toxin under GA with a supraglottic airway device without NDMR between November 2021 to December 2022 were prospectively recruited to record and study the eye movement and eye positions during both process of induction and intra-operatively after induction for any eccentric eye-movements. It is to be noted that patients were prospectively monitored from the start of induction because maximum anesthetic depth and its fluctuation occurs during induction and there would be greater chance to witness them. And the patients who witnessed down-rolling of eyes and/or downward eccentric eye positioning during induction or intra-operatively after induction were recruited in the study.

Exclusion criteria were patients with neurological disorders like cerebral palsy, seizure disorder, or paralytic or restrictive squints, patients with eccentric upward movement alone (without accompanying downward movement or down-positioning), and in cases without anesthetic details or details of intra-operative of eye movements.

### 2.3. Study methodology

Inhalational induction was started with 8% sevoflurane in a 50% oxygen/nitrous-oxide mixture (N2O) mixture and an intravenous line was placed both in prospective and retrospective cases. After attaining jaw relaxation, the supraglottic airway device (laryngeal mask airway) was inserted. Synchronous intermittent mechanical ventilation was started with pressure support on “GE Datex-Ohmeda Avance S5 (USA) Anesthesia machine.” Tidal volume, frequency, and pressure support was adjusted to achieve an end tidal carbon-dioxide (ETCO2) between 35 mmHg and 40 mgHg. Venous access was obtained once the child was sedated with inhalational induction. After establishing the intra-venous line, fentanyl (0.5–2 μg/kg) and propofol (if required) was injected as an inducing agent to increase depth of anesthesia. Once the laryngeal mask airway was secured, sevoflurane was reduced to maintain airway along with oxygen and nitrous oxide (50:50) without a muscle relaxant. Corresponding values of minimum alveolar concentration (MAC) (7) (as calculated by the anesthetic machine from ET sevoflurane of the anesthetic agent) at the time of onset of down drift and its centralization were noted along with their timings. This included data-points during induction (prospective data) and after induction during surgery (both retrospective and prospective data). MAC is defined as the concentration of inhaled anesthetic within alveoli at which 50% of people show immobility in response to any nociceptive stimulus (7). Also sequence of events prior and following downward eye movements in different cases were noted in both retrospective and prospective data-points. Timings of anesthetic agents, such as intra-venous agents like fentanyl and propofol (if given) during or after induction in relation to down-drifted eye movements, were noted. Types and duration of surgery were also noted. Any oculo-cardiac reflex if present and any change in heart rate and pupil size were also noted.

### 2.4. Recording of eye movement

Details of eye movement recordings (for the retrospective study) were available from our recorded intra-operative data base. Pre-operative recordings of GA induction were done using smart-phone cameras (I phone 13) for our prospective series. Eye movements and timings during induction were recorded and noted by the ophthalmologist starting from the point the child was sedated from the awake condition. The ophthalmologist continually monitored the eye positions by retracting both lids and opening the eyes together. Vitals and sevoflurane concentration (in terms of MAC) were simultaneously recorded from the monitor by separate observers from induction till the airway was secured by a laryngeal mask (Supplementary Video 1).

### 2.5. Scoring of eye movement

**Figure 1:**
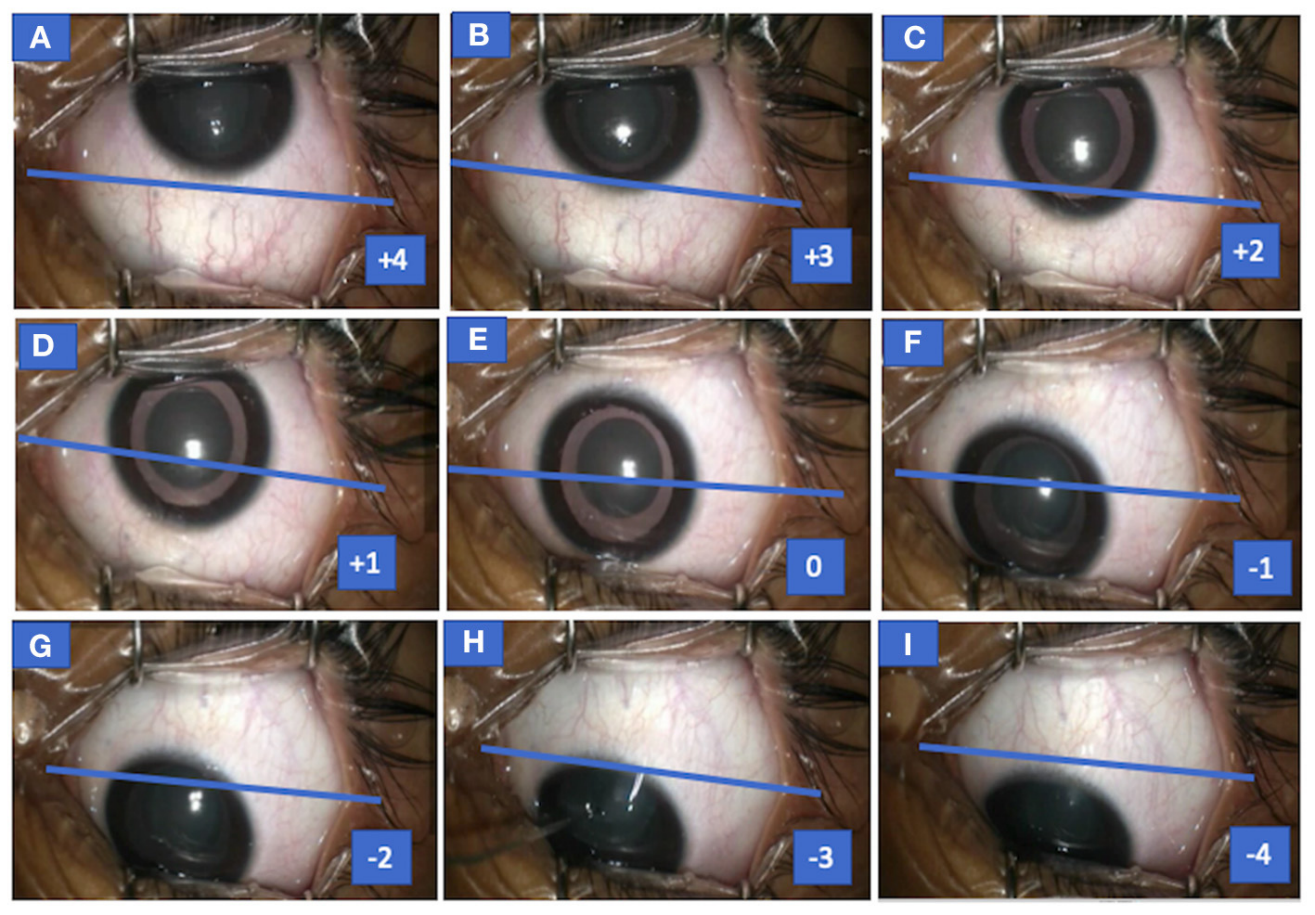
Photograph of the left eye of case 1 (congenital cataract) showing both up and down drift with scores for various eye positions. Eye seen in up-drifted position (+4) before the start of surgery (MAC = 1), following which sevoflurane concentration was increased and eye slowly drifted down (+4 to +1) **(A–D)**, became centralized momentarily **(E)** but overshot or drifted in downgaze **(F–I)**, and eye assumed an eccentric position in downgaze (−1 to −4) at MAC = 1.8. MAC, minimum alveolar concentration.

Records of eccentric down-positioning of eyes from the recordings were evaluated in both inferior positions of eyes (toward inferior fornix) in relation to the medial canthus; superior limbus/superior half of cornea was scored on an ordinal scale from −4 to 0 (Figure 1).

−4 = line joining medial and lateral canthi passes above the superior limbus

−3 = line joining medial and lateral canthi passes via superior limbus

−2 = line joining medial and lateral canthi passes between superior limbus to superior one-fourth cornea

−1 = line joining medial and lateral canthi passes between superior half to one-fourth cornea

0 = pupil position aligning medial canthus

Similarly, positive values (0 to +4) reflected superior positions of eyes (toward superior fornix) in relation to the medial canthus and inferior limbus or inferior half of the cornea.

### 2.6. Definition of eccentric eye movement and positioning

“**Eccentric eye movement**” is defined as the off-axis vertical movement of the eye from its central position in a quick-rolling/drifting fashion. “**Eccentric position**” is defined as off-axis vertically deviated positioning of the eye from its central position after the vertical eye movement. This eccentricity is either described as upward or downward depending on the movement/position of the eye toward the upper fornix or lower fornix respectively. In our study, the term “**drift”** and **“rolling**” have been interchangeably used for the upward and downward movements as these movements were smooth and slow similar to the drifts seen in dissociated vertical deviation or slow rolling eye movements seen during sleep onset/Bells phenomenon. Also, both terms describe sweeping eye movements with marginally slow speed (encountered in our study) which differentiates them from rapid eye movements/saccades of the awake state. An electronic search was performed using keywords: eye movement, general anesthesia, eccentric eye position, anesthetic depth, sleep, and anesthesia. The search of published literature for the review was made via PubMed, Med line, Google Scholar, and Ovid along with checking for cross-references.

### 2.7. Sample size calculation and statistics analysis

Sample size calculation was done using G^*^Power software (version 3.1.9.4). In order to get a statistically significant mean difference of 0.4 MAC (with SD of 1) between down-rolling and centralization at 80% power of study and 95% confidence interval, sample size for down-rolling events came out to be 52 patients. Anticipating a 10% iteration rate, we recruited 60 patients.

Data sets (events of EDEM/EDEP) were divided into two parts: (a) **during induction (DI)** from prospective data and (b) **after induction (AI)** from start to end of surgery (both prospective and retrospective data). The statistical analysis was carried out using Statistical Package for Social Sciences (SPSS Inc., Version 22.0. IBM Corp., Armonk, NY for Windows). Normality of quantitative data like age, HR, MAC, duration, down-rolled and up-rolled eye position score, etc. were checked by Shapiro Wilk test and Kolmogorov-Smirnov test of normality. Normally distributed quantitative variables were presented as mean and SD whereas skewed data were represented as median IQR (Interquartile range). Qualitative or categorical variables like gender, number of down and up movement, propofol given (yes/no), etc. were described as frequencies and proportions. Mean ± SD of MAC and median (IQR) of eye score both during down-rolling and centralization were calculated for both PI and AI data and mean difference and median difference of the two were calculated using paired *t*-test and Wilcoxon Signed Rank test respectively. Median difference of two independent variables (age of PI vs. DI data; eye scores of right vs. left eye, MAC during induction without propofol vs. with propofol before down-drift) were calculated by independent Mann Whitney U test.

Additionally, Median MAC (IQR) during up-rolling and its relationship with down-rolling were studied from AI data. All eye positions including up (4 to >0), central (0), and down (−4 to < 0) eye positions with various scores correlated were with DOA (in terms of MAC) for both AI and DI data via Spearman's rank correlation. ROC curve analysis was performed on both AI and DI data to find out MAC cut off value for down movement in two age groups: ≤ 2 years and >2 years. All inferential statistical tests were two-sided and were performed at a significance level of *p* < 0.05 with 95% confidence Interval (95% CI).

## 3. Results

**In retrospective (R) series**, a total of 48 patients were identified to have up-rolling/down-rolling eccentric eye movements or eccentric eye positions intra-operatively among a total of 249 children over 59 weeks. Among them, only 12 patients with downward eccentric position of eyes (with or without upward eccentric eye position) were enrolled. As two patients re-encountered tonic down-rolling during subsequent surgeries, **14** down-rolling events (**after induction**/**AI)** were included in the study for final analysis.

A total of 62/131 patients were included in the **prospective (P) series**, out of which data of **70** abrupt down-rolling events were populated (**8** events **after induction** from start to end of surgery and **62** events **during induction/DI** of anesthesia).

So, a total of **22 AI** (14 retrospective and 8 prospective) and **62 DI** were included in the final analysis (Table 1). The median age of 74 (62P+ 12R) patients was 2 years (IQR 1–5). There were 32 male and 42 female participants. HR (Median 120; IQR 110–124) was maintained throughout the event. The demographic profile of both retrospective and prospective data is shown in Table 1.

**Table 1:** Demographic profile of retrospective and prospective data.

	**Retrospective**	**Prospective**
Number of children analyzed	249	131
Number of patients witnessing down-drift	12	62
Number of down-drift events	14	74
M/F	8:4	24:38
Age	1.375 ± 0.85 years	3.52 ± 2.97 years
Type of ocular surgery	Phacoemulsification-3 Botox injection-8 Posterior capsulotomy-1 Squint Sx-2	Squint Sx-46 Botox injection-16
Distribution of EDEM/DP events	Before start of Sx-7 (2^*^) During Sx-6 (4^*^) End of Sx-1(0^*^)	Before Start of Sx-5 (3^*^) During Sx-2 (1^*^) End of Sx-1 (0^*^)

Sx, surgery; M, male; F, female; EDEM/EDEP, eccentric downward eye movement/eccentric downward eye-positioning.

^*^Refer to number of patients with up-rolling events preceding down-rolling among them.

### 3.1. Depth of anesthesia during down-rolling and centralization

#### 3.1.1. After induction (from start of surgery to end)

Mean MAC of EDEM/EDEP and centralization after induction in 22 events were 1.60 ± 0.25 and 1.18 ± 0.17, respectively, (*p* = 0.000) (Table 2).

#### 3.1.2. During induction

Mean MAC of EDEM/EDEP and centralization during induction in 62 patients was 2.19 ± 0.43 and 1.39 ± 0.26, respectively, (*p* = 0.000). Mean total time passed from start of stage 3 anesthesia (when child got unconscious) to start of down-drift event was 112.79 ± 36.21 s (Table 2).

#### 3.1.3. ROC curve for both DI and AI data

**Figure 2:**
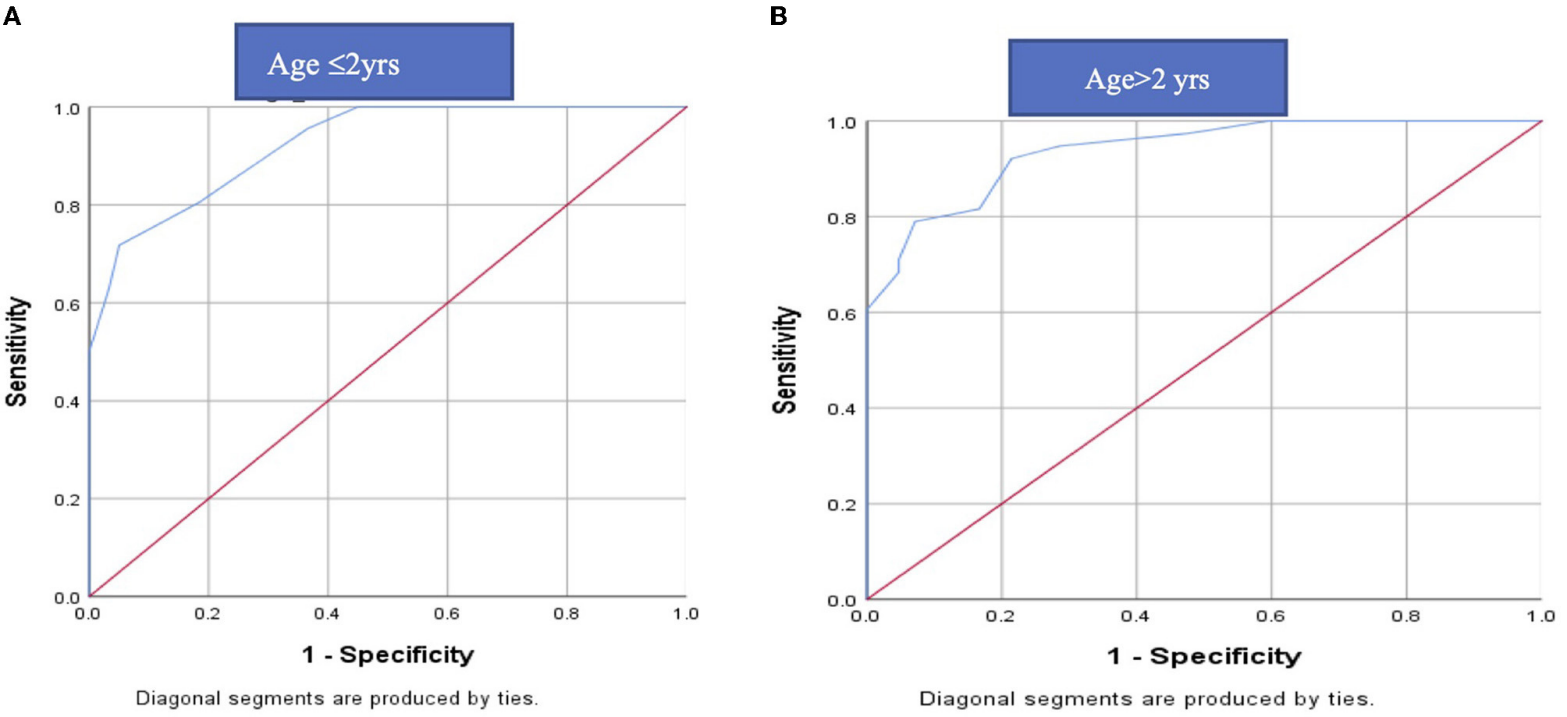
ROC curve of MAC for DEEM/EDP in **(A)** ≤ 2 years and **(B)** > 2 years: The cut off value of MAC for EDEM/ EDEP from ROC curve came out to be 1.65 in ≤ 2 years (71.7% sensitivity and 95% specificity) and 1.75 in >2 years (78.9%sensitivity and 92.9 % specificity). ROC, receiver operating characteristics; EDEM/EDEP-eccentric downward eye movement/eccentric downward eye-positioning; MAC-minimal alveolar concentration.

The cut off value of MAC for EDEM/EDEP from ROC curve came out to be 1.65 in ≤ 2 years (71.7% sensitivity and 95% specificity) and 1.75 in >2 years (78.9%sensitivity and 92.9 % specificity) (Figure 2).

**Table 2:** Details of data-sets both after induction (AI) and during induction (DI).

		**After induction/AI**	**During induction/DI**	
No of patients analyzed		131 (P)+245 (R) = 376	131 (P)	
No of down-rolling events (patients enrolled)		8 (P) + 14 (R) = 22	62 (P)	
Number of up-rolling events preceding down-rolling		12 (6P+6R)	54	
Median Age (IQR) in years		1.05 (IQR 1–1.5)	3 (IQR 1.06–6)	
		*p =* 0.001		
DOA (Mean MAC)	During start of induction	-^*^	2.65 ± 0.48	
	During down-roll	1.60 ± 0.25	2.19 ± 0.43	
	centralization	1.18 ± 0.17	1.39 ± 0.26	
	Up-rolling	1 (IQR 0.98–1.1)	NA^**^	
Mean MAC (IQR) of down-rolling events witnessed immediately after propofol (*n =* 20)		-^***^	2.0 (1.63–2.3)	*p* = 0.022
Mean MAC (IQR) of down-rolling events witnessed immediately without/before propofol (*n =* 42)			2.25 (2–2.6)	
Recurrence of down-rolling event in same patient		2	2	
Timing (median, IQR)	Time elapsed during induction from the time child loses consciousness (stage 3) to start of down-rolling mean	^*^	112.79 ± 36.21 s	
	Total time taken in down-rolling from centralized position	10 (8–14) s	9 (8–12) s	
	Total time taken in centralization from down-rolled position	85 (60–117) s	120 (80–165) s	
	Time elapsed from propofol injection to down-rolling of eyes (*n =* 20)	^***^	10 (8–12) s	
pupil size during down-rolling		2.5(IQR 2–3.5) mm	2(IQR 1.5–2.5) mm	
Eye score	Down-rolling	−3 (IQR −2 to −4)	−3 (IQR −2.5 to −4)	
	Number of asymmetricity	4/22	23/62	
	Up-rolling	2 (1–2.25)	NA^**^	
correlation between eye position (scores −4 to +4) and DOA (MAC)		−0.714; *p =* 0.000	−0.832; *p =* 0.000	

P, prospective; R, retrospective; M, minimal alveolar concentration; DOA, depth of anesthesia; EDEM/EDEP, eccentric downward eye movement or eccentric downward eye-positioning, IQR, interquartile range.

^*^Not part of inclusion criteria.

^**^NA-not applicable as up-rolling of eyes occur transiently during induction, and MAC value at the time of up-rolling do not denote the corresponding depth of anesthesia and hence eye score of up-rolling for induction was not measured.

^***^Propofol was not given.

### 3.2. Correlation between DOA and eye position

The median (IQR) eye score at the time of down-drift and correlation between MAC (DOA) and various eye position (scores −4 to +4) for both **AI** and **DI** data-points is shown in Table 2. All eyes were slightly adducted during eccentric positioning in down gaze (opposite of upward and outward positioning in up-gaze). Strong negative correlations between MAC (DOA) and various eye positions for both AI and DI data have been shown in Table 2.

### 3.3. Age and DOA during EDEM/EDEP

Median age of AI and DI have been shown in Table 2. Their median difference was statistically significant (*p* = 0.001).

### 3.4. Time duration of down-rolling and return of down-drifted to the central position

#### 3.4.1. After induction

Eccentric down-positioning of eyes partially interrupted the ongoing surgical procedure. When down-positioning of eyes was encountered, anesthetists usually decreased the DOA slightly, on a trial basis toward the recommended lower limit by decreasing the volatile agent. Eccentric downward eye position was maintained till anesthetic depth was slightly lightened when eyes returned to their resting position. All the movements were smooth throughout. Tonic downward movement was quick but the return was comparatively slow and variable depending on varying rates at which the individual anesthetist adjusted DOA and how fast MAC was changed. Median time taken in down rolling from centralized eye position was 10 s (IQR 8–14) and time taken in centralization from eccentric downward position was 85 (IQR 60–117) s (Table 2).

#### 3.4.2. During Induction

Median time taken in down rolling from centralized eye position was 9 (IQR 8–12) s and time taken in centralization from eccentric downward position was 120 (IQR 80–165) s (Table 2).

### 3.5. Timing of onset of down-rolling and its relationship with upward drift

#### 3.5.1. After induction: down-rolling/positioning of eyes was seen either (a) with upward drift or (b) without upward drift

**(a)** In 10 events **(4P+6R)**, downward drift during surgery was preceded by an **upward drift** (Table 1). In five events, eyes were found up-rolled **before the start of surgery** after cleaning, draping, and using an eye speculum (Figure 1; Supplementary Video 2) and in five events, patients encountered up-rolling after the start of surgery with application of noxious stimuli (e.g., traction, during conjunctival incision). This upward drift was seen when the DOA was on the borderline lighter side in all cases. Documented median (IQR) MAC in 10 cases at the time of upward drift was **1 (IQR 0.98–1.1)** (Table 2). When anesthetists were informed of the up-rolling of eyes, increase in sevoflurane concentration (increasing MAC) was used as a measure to resolve the problem in all cases. Within a few minutes of increasing MAC, the eye returned to its primary position but, as the procedure was about to be re-started, eyes overshot in down gaze at the same time (Figure 1; Supplementary Video 2).

**Figure 3:**
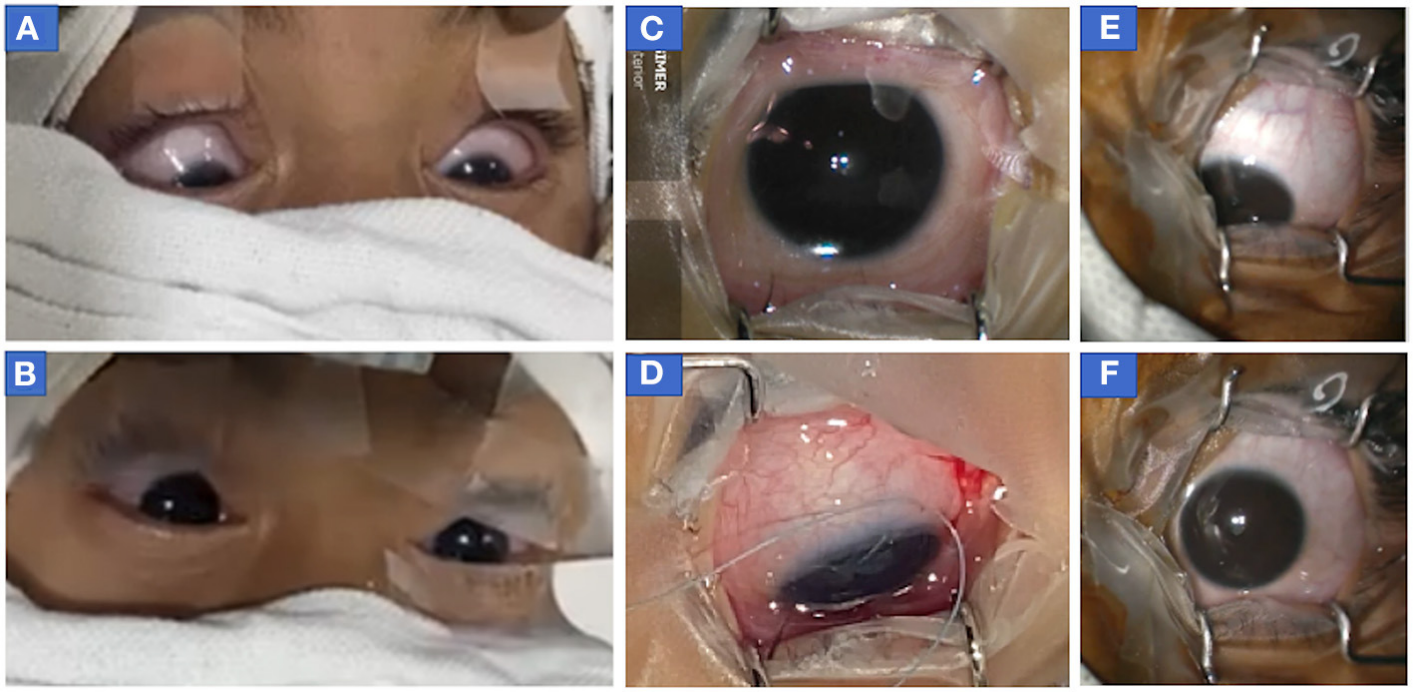
Photograph of eyes of patients who showed downdrift alone without updrift. **(A, B)** Both eyes of case 2 (before botulinum injection) showed **(A)** symmetric down-positioning of eyes before the start of surgery (MAC 1.4) when eyes were cleaned, draped, and opened following which **(B)** sevoflurane concentration was decreased and eyes returned to the central position (MAC = 1.2) within a minute of decreasing sevoflurane concentration. It should be noted that recorded values of MAC at the time of intubation were 1.7 and MAC was stabilized to 1.4 within a few minutes of decreasing sevoflurane concentration after intubation when eyes were opened to see their position at the time of cleaning and draping. **(C, D)** The right eye of patient 2 (before second botulinum injection) at 14 months of age in the central position (MAC = 1) at the time of the start of surgery **(C)** but as anesthesia was deepened (to improve oxygen saturation as patient was not able to maintain spontaneous breathing), eye turned in and down **(D)** after temporary release of muscle traction during surgery following passage of traction suture, conjunctival incision, and hooking of muscle. (at MAC = 1.4) **(E, F)** Photographs of eccentric eye positioning in downgaze with slight adduction (toward the nose) in left eye before start of surgery in case 3 (infantile esotropia) (MAC = 1.6) **(E)** and after eye achieved centralized position when the depth of anesthesia was decreased (MAC 1.3). MAC, minimum alveolar concentration.

**(b)** Downward eye eccentric position of eyes **without upward drift** was seen in 12 patients (8R+4P; Table 1). Seven events of downward eccentric down-positioning were encountered **before the start of surgery** after cleaning and draping when eyes were found eccentrically down-positioned (Figures 3A, E; Supplementary Video 3). Three events occurred **after the start of surgery** during intermittent withdrawal of traction in eye (at the same MAC, when eyes were central and surgery was started; Figure 3D). Eyes also down-rolled during important steps in a few patients e.g., just before re-introducing phacoemulsification probe, injection of Botulinum toxin in medial rectus etc. but fortunately no complications were encountered as during these movements, no instruments were close to important structures of the eye. And two events of downward rolling were witnessed in a patient **at the end of surgery** (Supplementary Figure 5; Supplementary Video 4).

When the surgeons tried to manually rotate the eyes to the central position, difficulty was felt as the tonic downward force was experienced by the surgeons in all cases. The surgical procedure was abandoned for a few minutes as in the presence of an eccentric eye position the continuation of the surgical procedure became unexpectedly difficult.

#### 3.5.2. During induction

At the time of induction all patients witnessed transient up-rolling and outward drift following which down-rolling was witnessed in all cases. But MAC during transient up-rolling in DI data was not analyzed because it was witnessed transiently during beginning of induction when sevoflurane flow was high.

### 3.6. Relationship of downward drift with anesthetic agent used

#### 3.6.1. After induction

21 episodes of down-rolling which occurred during surgery were maintained on sevoflurane. Only one patient who witnessed down-rolling during switching of sevoflurane to isoflurane (when anesthetic concentration inside lungs became momentarily high) was also included in the study. None of the patients were injected with any intra-venous anesthetic agent (like propofol) prior to witnessing a down-rolling event.

#### 3.6.2. During induction

Induction was performed with sevoflurane and fentanyl in all cases. Propofol was given in 28 out of 62 patients among which 20 patients experienced episodes of down-rolling immediately after propofol injection (median time 10 s) while down-rolling event was noted prior to propofol injection in eight patients. MAC at which downdrift was witnessed immediately after propofol injection was lower compared to MAC at which downdrift was witnessed without/before propofol injection (*p* = 0.022; Table 2).

### 3.7. Relationship of repeated anesthetic exposure on the incidence of downdrift

#### 3.7.1. After induction

15 patients underwent repeat surgeries under GA without NDMR out of which recurrence of event was seen in five patients.

### 3.8. Relationship of pupil size with EDEM/EDEP event

No change in pupil size was noted during EDEM/EDEP in both AI and DI data.

#### 3.8.1. After induction

Proparacaine was given to all patients before the start of the procedure. The median pupil size during EDEM/EDEP was 2.5 mm (IQR 1.5–3.5).

#### 3.8.2. During induction

In four patients (three pediatric cataracts and one pseudophakia with posterior capsular opacification), change in pupil size could not be estimated as pupils were pharmacologically dilated for the surgical procedure. In the remaining 18 patients, the median pupil size during EDEM/EDEP was 1.75 mm (IQR 1.5–2 mm).

### 3.9. Symmetry of eye position between two eyes

#### 3.9.1. After induction

**Figure 4:**
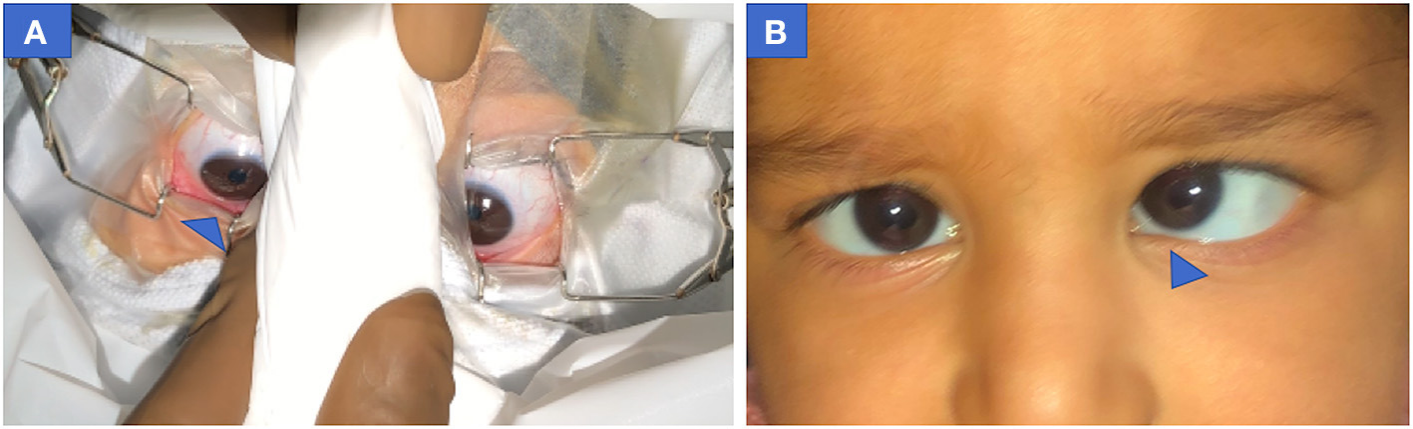
Pre-operative photograph of both eyes showing asymmetric down-positioning of eyes in case 4 (infantile esotropia) **(A**) Right eye more down-positioned than left eye at MAC = 2. **(B)** Pre-operative clinical profile of same patient showing esotropia with left eye Dissociated vertical deviation. MAC, minimum alveolar concentration.

Downward drift was asymmetrical in four patients (Figure 4A). Pre-operative records of one patient revealed eye dissociated in a vertical deviation (Figure 4B).

#### 3.9.2. Before induction

Mild asymmetry in downward drift was seen in 23 patients during down-drift and in extreme eccentric down-drifted position. The median eye score was −2.68 ± 0.99 in the right eye and −2.87 ± 1.01 in the left eye in 23 patients (*p* = 0.939).

## 4. Discussion

### 4.1. Depth of anesthesia during down-rolling and centralization

Our study showed that, in absence of muscle relaxant, down rolling of eyes occurred at relatively deeper depths of sevoflurane anesthesia compared to anesthetic depths at which eyes were centralized. This was demonstrated by the higher value of MAC for EDEM/EDP relative to centralized position (*p* = 0.001) in all cases (both DI and AI data; Table 2). Results of our study matched the finding of Kook's study which mentioned downward eye drift with BIS values below 35 or deep anesthesia (4). Anesthetic depths (MAC) at which these fluctuations in eye position appeared was within permissible anesthetic limits of induction (8) as well as surgery (stage 3 anesthesia). Inhalational agents are known to cause a dose-dependent suppression of reflexes and movements (6). It is said that a complete lack of electrical discharge in the extraocular muscles occurs during deep sleep or deep anesthesia, (6) but our study confirmed that the increasing DOA may not always be effective in centralization of eyes or in reducing eye movements (9).

### 4.2. Correlation between DOA and eye position

A significant negative correlation of eye score (-4 to +4) with DOA signified that eyes up-rolled in lighter planes, (4) centralized on slight increase, and down-rolled on further deepening the DOA (4). In his study of 32 patients, Kook et al. (2) scored the vertical position of each eye on an ordinal scale from −2 to +6, according to its height in relation to the medial canthus, and studied the relationship between fixed eccentric eye elevation and DOA during surgery. He encountered elevation of the eyes in 83% and downward position of eyes in 2% (one child) with BIS values < 35, probably because he maintained a lower concentration of sevoflurane for induction. We did not include BIS in our study because the majority of our children were younger than 2 years old and values of BIS in infants and young children do not indicate a similar concentration of sevoflurane like adults and hence may not be always reliable (10). Lower values of BIS generally indicate deeper levels of anesthesia which can also be corroborated from the higher values of MAC used in our study.

### 4.3. Relationship of Age with DOA during down-drift

Average age of children who witnessed a down-rolling event during surgery was lower compared to the age of children witnessing down drift during induction (Table 2). It could be because infants and smaller children are more susceptible to fluctuations in DOA and a higher depth is reached with lower MAC in a very short time. This is also supported by the lower cut off level in infants and younger children than older children shown in ROC curve in our study. Reasons for greater down-drifts in smaller children could be because of the (1) lower threshold for sevoflurane, (2) start of sevoflurane induction prior to placement of intravenous line (as injection of intra-venous agents not possible without sedation) and (3) extended period of high sevoflurane flow due to difficult cannulation in small children. Intravenous agents which are injected in co-operative older children by placing an intra-venous line before starting sevoflurane increase depth of anesthesia and decrease the requirement of sevoflurane. Few older children also witnessed down-drift during induction in our study. Hence the results of our study showed that even though smaller children are more susceptible, there remains risk to older children as well specially with high flow of sevoflurane (high MAC) for prolonged periods like delay in intra-venous cannulation etc.

### 4.4. Time duration of down-rolling, downward eye positioning, and return of down-drifted to the central position

Our study showed that these movements are swift and the ophthalmologist has to be careful to identify such movement and withdraw instruments carefully if such movements are encountered. Also, the anesthetist must avoid fluctuation in DOA in short surgeries without NDMR and should be aware that eyes shall remain in this down-rolled position till sevoflurane flow, and in turn anesthetic depth, is decreased.

### 4.5. Timing of onset of down-rolling during GA

#### 4.5.1. During induction

In his study under different depths of general anesthesia, Power et al. (9) observed final eye position in downward direction after induction in a few patients but details including timing, number of down-rolling events, and DOA were never described. Our P-study showed that tonic EDEM/EDEP occurred during induction in 62 out of 132 patients. Such high numbers of down-rolling were witnessed during induction in our study because in general, induction with sevoflurane is rapid (alveolar concentration approaches inspired concentration much more rapidly) and because its flow to lungs is generally kept high during induction. The mean concentration of sevoflurane during induction at which EDEM/EDEP occurred prior to LMA insertion was high (median MAC = 2.65; IQR 2.28–3.0) which matched with other studies (8). In children, where it is common to use a volatile induction, sevoflurane is the preferred choice. In a busy tertiary center like ours with high volume cases it is not uncommon to see reduction of the time for inhalational induction by using a concentration in excess of 1 MAC to speed-up induction as it would take some time for the patient to go to sleep if 1 MAC of volatile was used to induce anesthesia. It is then slowly decreased to the desired level once the child is cannulated and other intra-venous anesthetic agents are given to increase depth of anesthesia which decreases requirement of sevoflurane flow. This is when the supra-glottic device is inserted and then lower flow is sevoflurane is maintained while eyes are centralized and the child is handed to the ophthalmic surgeon.

#### 4.5.2. After induction

Quick induction and failure to lower sevoflurane (below the threshold value for that age group) explains why few children witnessed downward eye-positioning following handing over to the ophthalmic surgeon as it was the extension of down-rolling events during induction which was evident when eyes were opened after cleaning and draping before the start of surgery. Power et al. (9), while studying the DOA in young adults via sevoflurane induction, compared eye signs with EEG polysomnography and showed that the deepest level of sleep was reached on average 3 min before the onset of eccentric ocular positioning, thereby suggesting that eccentric eye movements may occur even when a patient appears satisfactorily anesthetized. This also points toward an understandable lag between anesthetic depth reached at the level of lungs and its effect seen at the level of CNS (eccentric eye positioning). This could be why many of our patients (*n* = 11) experienced downward movement **before or at the start of surgery** because of the lag between change in anesthetic depth (increase) after induction and start of surgery which might overlap as most of the children witnessed downward movement before/at the start of surgery, depending on DOA and the child's threshold value.

Some children witnessed downward rolling **in the middle or at the end of surgery** potentially because DOA (although MAC was kept constant) varies with the level of stimulus (like traction on eye) the patient is experiencing. This may be illustrated by the fact that one of our patients experienced downward movement at the end of surgery (during conjunctival suturing) on the same MAC eyes were central and was being operated upon. Oculo-cardiac reflex was noticed in two cases following which depth of anesthesia was increased which in turn caused the down-rolling event in one patient.

### 4.6. Up-rolling and its timing with down-rolling

Many children experienced down-rolling during surgery immediately after up-rolling (Table 1). In our case series, upward drifting of the eyeball was also seen under lighter planes of anesthesia during surgery in response to any noxious stimuli in the form of pressure on the globe or any manipulation of the eyeball as demonstrated by lower MAC values (median 1.0) (4, 11). Though literature describes slight up-rolling of eyes with the cessation of voluntary eye movement as the point of sufficient anesthetic depth, (12) ocular surgeries are not performed under this anesthetic depth. Rossiter et al. (3) reported its substantial increased incidence without the use of muscle relaxants. Harrad and Stoddart suggested that Bell's phenomenon, a natural protective reflex, which occurs both in the awake state and with lighter planes of anesthesia (though the patient is not awake), may explain the entity (11, 13). The exact neural mechanism is unknown but involves brainstem pathways between the seventh cranial nerve nucleus in the pons and the third cranial nerve nuclear complex in the rostral midbrain. Hiraoka et al. (14) have suggested that the mesencephalic reticular nucleus may play an important role in integrating these two patterns of movement (bilateral lid closure and upward movement of both eyes).

Bell's reflex is extinguished with deep planes of anesthesia, such that the eye remains in the neutral gaze (13, 15). And this upward drift triggered by traction during surgery prompted the anesthetist in our study to increase the DOA to make the eyes return from the upward position following which tonic down shoot of eyes was encountered. This was seen in a few cases and could be because of the attempt to rapidly increase the flow of sevoflurane and rapidly deepen/optimize the anesthetic depth which crossed the threshold for that child.

### 4.7. Relationship of down-rolling with propofol

#### 4.7.1. During induction

Propofol was given in 28/62 patients in which a down-rolling event was seen immediately after injection in 20 children. Time interval elapsed from propofol injection to down-drift (10 s) matched with the onset of action of propofol (16) (Table 2). Median MAC during which EDEM/EDEP was witnessed immediately after propofol was slightly lower than MAC at which down-rolling was experienced without propofol (Table 2), thereby suggesting a synergistic effect of high sevoflurane flow and propofol in increasing DOA and causing sudden EDEM/EDEP. The remaining 72 patients who did not experience down-rolling and hence were not recruited in the study may have received a low flow of sevoflurane (low MAC during induction) from the start due to quick cannulation or prior injection of propofol and fentanyl before starting induction in co-operative older children.

### 4.8. Relationship of repeated anesthetic exposure to the incidence of downdrift

Our study showed that the same patient may or may not experience such events again and it depends on sevoflurane flow used during induction and maintained in surgery, threshold for that age, and rapidity at which DOA is changed.

### 4.9. Relationship of pupil size with anesthetic depth

No change in pupil size (or no dilatation of pupils) occurred throughout the study whether the eyes were centralized or down-rolled, which implies MAC at which down-rolling occurred (highest depth of anesthesia in our study) was also within permissible limits of surgical anesthesia (stage 3) (8). Our study showed that eyes rolled up in the lighter plane of stage 3 anesthesia and down-rolled during deeper planes of stage 3 anesthesia without muscle relaxant. So, there is a **narrow band of anesthetic depth** in which ocular surgery has to be performed. If anesthetic depth goes too low eyes shall roll up and if it gets too deep eyes shall roll down.

### 4.10. Symmetry between two eyes

Our study showed eyes generally move symmetrically in a downward position but can move asymmetrically (Figure 4). In a majority of cases the difference remained insignificant. Though we could not understand the exact reason for asymmetry, we suspected asymmetric dissociated vertical deviation in the child to alter the eye position in two cases.

### 4.11. Probable pathophysiology behind downward drift

The pathophysiology of down-rolling under GA is unknown. We tried to hypothesize based on the shared neurochemical and behavioral features of sleep and general anesthesia (17, 18). Various anesthetics have been demonstrated to alter brain systems involved in sleep-wake control (17–19). Many medicines produce general anesthesia that is very comparable to NREM sleep, (20) including a breakdown in efficient cortical communication (21, 22) and inactivation of the thalamus and midbrain reticular formation along with loss of awareness (23). Anesthetized patients' brains may be trapped in an NREM-like state, preventing access to REM sleep and waking (22, 24). In a wakeful state, the mesencephalic reticular development is critical in creating a vertical saccade (25). REM sleep activates cholinergic neurons in the reticular formation (26). The similar velocity-amplitude correlations of rapid eye movements during REM sleep and spontaneous saccades in the dark when awake suggest a shared neural circuit (27). Since eye movements changed with MAC, we hypothesize that a sudden increase in anesthetic concentration (which might have been potentiated with use of prolonged high sevoflurane flow during induction for deepening DOA) caused temporary irritative effects in the central nervous system, causing temporary switching (28) of non-REM to a REM sleep-like state or isolated REM sleep-like traits being expressed during non-REM sleep-like state (29) with activation of the mesencephalic reticular formation and neurons.

The irritative effect of increased anesthetic concentration can be many-fold, although prospective studies are required to evaluate these theories.

1. An increase in sevoflurane concentration (higher MAC) and differential sensitivity of cortical and subcortical areas to sevoflurane concentrations (30). In adults, Mourisse and colleagues (31) found that the blink reflex (brainstem function) was more susceptible to sevoflurane than BIS (forebrain function). The interstitial nucleus of Cajal, the mesencephalic reticular formation, and the posterior commissure are all located at the meso-diencephalic junction (32). Forced downward gaze is prominent in this area's lesions, indicating a vertical gaze plane imbalance (33). In our cases, down gaze neurons in the midbrain were possibly selectively irritated by high flow of sevoflurane (34, 35). Younger children's higher sensitivity to sevoflurane for specific subcortical areas (i.e. mesencephalic control) than older subjects may explain its greater prevalence only in younger children (30) when the threshold was crossed. Different flow rates of anesthetic agent used, different time taken, and different DOA achieved during intubation as well as different DOA (MAC value) on which the patient was stabilized before handing them over to the ophthalmic surgeon for the procedure could be factors influencing the non-occurrence of eye movements during repeat exposures in the same patients.
2. Another potential reason could be related to shortened autoregulation and vasodilatory impact of sevoflurane generating transient reduced cerebral blood flow (CBF) and an irritative effect which reverses when concentration is decreased (36, 37). The lower limits of sevoflurane autoregulation are close to young children's basal mean arterial pressure (38). The fact that our patients' exhibited eye movements beyond 1.5 mean MAC supports this hypothesis, as CBF remains unchanged by sevoflurane up to 1.5 MAC value (36, 39). It is possible that the CBF sensitivity window is quite narrow and specific to each individual. It is likely that the narrow autoregulation limit was violated when the depth went above the child's acceptable limit. The posterior circulation zone supplying the mesencephalic-diencephalic junction comprises structures critical for vertical sight and vergence, (40) and may be transiently impaired, manifesting eye movements. The concentrations at which these downward movements were seen in our cases do not necessarily lead to cerebral ischemia in healthy children. As thousands of sevoflurane inductions are performed every day in children, and neurologic complications are quite rare. The movement occurred only when the MAC level was below or beyond the narrow limit in sensitive children.
3. A third possibility is that sevoflurane is known to excite the neurons in Locus Coeruleus (LC) (41). The LC is a pontine nucleus, with the largest group of noradrenergic neurons in the brain, and is responsible for the tonic maintenance of the wakeful state (42). This nucleus has very widespread projections to cortical and subcortical regions and to the spinal cord (43). In addition, it also projects to the oculomotor nucleus which has been shown to have a high density of α1-adrenoceptors (44, 45). It is plausible that sevoflurane, at higher concentrations, induces the activation of the LC leading to the tonic contraction of the muscles innervated by the oculomotor nerve. As the LC is also involved in maintaining the wakeful state, once the DOA is on the lighter side it is possible that the same scenario of LC activation repeats itself. LC activity, through effects on α2-adrenoceptors in the Edinger-Westphal Nucleus (46, 47), can inhibit pupillary constriction by attenuating the light reflex. This might be the reason why we noticed ocular movement without any pupillary change. The interindividual differences in the specificity of these projections, in addition to the fact that MAC per se has been defined for producing immobility to surgical stimulus in 50% of the population, (48) may explain why these ocular movements are not noticed in every case.

The limitations of our study are the small sample size and absence of EEG monitoring in participants. The sample size limits generalization of reported median MAC values and the usefulness of the association between DOA (MAC value) and eye-positioning score. The sevoflurane flow was changed by anesthetist as per requirement in a child for quick induction which might have influenced the eye movements. Separate studies are required to be done with and without propofol to look for the impact on eye movements. Also, more research is needed on the link between eccentric downward movement, eccentric upward movement, level of anesthesia (using BIS and MAC values), and electrical activity of brain (Electro-encephalogram). In addition to electro-oculogram, we need to explore the tonic force in the inferior rectus to understand the pathophysiology of these eye movements, giving clues into ongoing subcortical processes. Further research is required on the entity's link to anesthetic depth and age. It will be fascinating to see if an anesthetic drug plays a role in halting these motions.

## 5. Conclusions

Our study concludes that sudden tonic down-drifts of eyes can occur in children without NDMR with the deeper plane of stage 3 sevoflurane anesthesia compared to depths at which eyes are centralized though both depths are adequate and under safe limits of anesthetic depth. This article intends to educate ophthalmologists and anesthetists about this unexpected eye movement during general anesthesia. Knowledge of this is important in short ocular procedures or surgeries under GA, especially without NDMR. In intra-ocular surgeries involving critical steps, muscle relaxants may be used for novices. Our study demonstrates the importance of keeping a stable DOA throughout and avoiding fluctuations. In the event of eye movement, the anesthesia team should be contacted immediately and the surgery should be restarted after the eye movements have stopped, and the eyes have returned to their centralized position.

## Data availability statement

The original contributions presented in the study are included in the article/Supplementary material, further inquiries can be directed to the corresponding author.

## Ethics statement

The studies involving human participants were reviewed and approved by Institutional Ethics Committee of the Postgraduate Institute of Medical Education and Research, Chandigarh, India. Written informed consent to participate in this study was provided by the participants' legal guardian/next of kin. Written informed consent was obtained from the minor(s)' legal guardian/next of kin for the publication of any potentially identifiable images or data included in this article.

## Author contributions

ShC, SLS, SwC, and JR contributed to conception and design of the study. ShC, JS, and SwC organized the database. ShC wrote the first draft of the manuscript. SLS and VG wrote sections of the manuscript. All authors contributed to the article and approved the submitted version.
