# Supplementary material for: Tonic down-rolling and eccentric down-positioning of eyes under sevoflurane anesthesia without non-depolarizing muscle relaxant and its relationship with depth of anesthesia
Source: Front Med (Lausanne). 2023 Jun 15;10:1029952. doi: 10.3389/fmed.2023.1029952 (PMC10311215; doi:10.3389/fmed.2023.1029952)
Supplement: Supplementary file 5 [file Data_Sheet_1.pdf]

Supporting information (video files) captions

Video 1: Data Recording of EDEM/EDEP During induction (DI): Left video shows manually everted lids from the time child (case1) was sedated on 8% sevofluorane in a 50% oxygen/nitrous-oxide mixture (N2O) mixture with simultaneous recording (right video) of parameter (MAC) on GE Datex- Ohmeda Avance S5 (USA) Anesthesia machine.

Note at the start of video child eyes are in up-gaze and divergent (0.0 seconds). MAC fluctuates from 2.5-2.8 at the start when while start centralizing at 0.39 seconds but soon left eye start overshooting in downgaze at 0.43 seconds (at 2.9 MAC) followed by right eye. Left eye completely **overshoot in downgaze at 1 minute (while MAC was 2.9)** followed by right eye at 1.21 seconds at 2.9 MAC.

Then MAC rapidly decreases to value 1.4 from 1.30 minutes to 2 minutes but eyes remain down-rolled till 2.52 minutes when eyes start up-rolling from down-drifted position and **centralizes at 3.23 minute (at 1.4 MAC).** Then sevofluorane was put off and Laryngeal mask was introduced.

Note was made through out recording time at which intra-venous line which was secured before or after injection of intravenous agent (here it was at 2.15 minutes when propofol and fentanyl was given after start of down-drift).

EDEM/EDEP- eccentric downward eye movement/eccentric downward eye positioning; MAC- minimal alveolar concentration
