# Supplementary material for: Tonic down-rolling and eccentric down-positioning of eyes under sevoflurane anesthesia without non-depolarizing muscle relaxant and its relationship with depth of anesthesia
Source: Front Med (Lausanne). 2023 Jun 15;10:1029952. doi: 10.3389/fmed.2023.1029952 (PMC10311215; doi:10.3389/fmed.2023.1029952)
Supplement: Supplementary file 6 [file Data_Sheet_2.pdf]

Supporting information (video files) captions

Video2: Video shows upward drifted position of the left eye found at the start of surgery (phacoemulsification) in a case 2 (at minimal alveolar concentration or MAC=1). On making anaesthetist aware of the up- drifted eye position, sevoflurane was increased and as soon as anaesthetic depth was increased, eyes drifted down but over-shot in extreme down-gaze. Noted MAC at the time of down- ward positioning of eyes was 1.8. The position lasted till the time depth of anaesthesia was lightened up to MAC 1.4
