# Supplementary material for: Tonic down-rolling and eccentric down-positioning of eyes under sevoflurane anesthesia without non-depolarizing muscle relaxant and its relationship with depth of anesthesia
Source: Front Med (Lausanne). 2023 Jun 15;10:1029952. doi: 10.3389/fmed.2023.1029952 (PMC10311215; doi:10.3389/fmed.2023.1029952)
Supplement: Supplementary file 7 [file Data_Sheet_3.pdf]

Supporting information (video files) captions

Video 3: Video shows case 3 left eye in eccentric inward and downward position in inferior fornix such that only the upper two-thirds of the cornea was visible at the time of the start of surgery (botulinum toxin injection in medial rectus) after cleaning and draping. The anaesthetist was informed and passive force was manually applied to rotate the eye to a central position but the strong tug was felt and the eye could not be manually manipulated. MAC at the time of this eccentric eye movement was 1.6. Anaesthetist rapidly decreased sevoflurane flow rate and eye smoothly descended upward and the central position was achieved (MAC 1.4) in 1 minute 10 seconds after which surgical procedure was completed.
