# Supplementary material for: Tonic down-rolling and eccentric down-positioning of eyes under sevoflurane anesthesia without non-depolarizing muscle relaxant and its relationship with depth of anesthesia
Source: Front Med (Lausanne). 2023 Jun 15;10:1029952. doi: 10.3389/fmed.2023.1029952 (PMC10311215; doi:10.3389/fmed.2023.1029952)
Supplement: Supplementary file 8 [file Data_Sheet_4.pdf]

Supporting information (video files) captions

Video 4: Video shows case 4 right eye in an eccentric downward position in the inferior fornix and then eye slowly rolled back to the central position from downward position. The recorded bi-spectral index (BIS) and minimal alveolar concentration (MAC) value at the time of this downward position were 45 and 1.7 respectively. Return eye movement was slow and smooth over few minutes (1 minute 40 seconds) when the depth of anaesthesia was optimized/lightened (1.3MAC).

Eccentric position in downgaze following tonic hypo-tropic movement was preceded by a slow upward drift of eye at the time of the last step of suturing the conjunctiva in the right eye. At the time of upward drift recorded BIS and MAC value was 55 and 1.1 respectively. On being informed anaesthetist increased the depth of anaesthesia by increasing sevoflurane concentration. Within a few minutes of increasing sevoflurane concentration, the eye returned to a primary position. But as the suturing was re-started, eyes quickly overshot in downgaze and assumed an eccentric position in downgaze (start of video).
