# Supplementary material for: Tonic down-rolling and eccentric down-positioning of eyes under sevoflurane anesthesia without non-depolarizing muscle relaxant and its relationship with depth of anesthesia
Source: Front Med (Lausanne). 2023 Jun 15;10:1029952. doi: 10.3389/fmed.2023.1029952 (PMC10311215; doi:10.3389/fmed.2023.1029952)
Supplement: Supplementary file 9 [file Data_Sheet_5.pdf]

Supplementary figure legend

e-Figure 5. Photograph of eyes of patients who showed downdrift at the end of surgery. **(A)** Right eye of patient 5 (1st event) showing downward eccentric eye position during suturing of conjunctiva (after injecting botulinum injection in hooked medial rectus with a small conjunctival incision) and **(B)** centralized eye position when MAC was lowered (DOA was lightened)**. (C)** Both eyes of same patient showing down-rolled eye position during subsequent squint surgery (4 muscle surgery) after effect of NDMR waned off and **(D)** centralized eye position after lightening depth of anesthesia.

MAC- minimum alveolar concentration; NDMR-non-depolarizing muscle relaxant; DOA-Depth of anesthesia
